# Electrical stimulation plus biofeedback improves urination function, pelvic floor function, and distress after reconstructive surgery: a randomized controlled trial

**DOI:** 10.1007/s00384-023-04513-7

**Published:** 2023-09-11

**Authors:** Aiming Lv, Tianzi Gai, Sichen Zhang, Qing Feng, Ye Li

**Affiliations:** Department of Obstetrics and Gynecology, Beijing Hospital, National Center of Gerontology, Institute of Geriatric Medicine, Chinese Academy of Medical Sciences, Beijing, People’s Republic of China

**Keywords:** Electrical stimulation, Pelvic floor muscle, Pelvic organ prolapse, Biofeedback, Electromyography

## Abstract

**Purpose:**

This study is aimed at assessing the effect of postoperative electrical stimulation (ES) plus biofeedback therapy on patient rehabilitation after pelvic floor reconstructive surgery.

**Methods:**

Patients with pelvic organ prolapse (POP) who had received pelvic floor reconstructive surgery were randomly allocated to the intervention group and the control group at a 1:1 ratio. Patients in the control group received routine postoperative nursing care. Patients in the intervention group underwent ES plus biofeedback therapy. The outcomes included the recovery of urination function, the improvement of pelvic floor muscle (PFM) strength, and the change of Pelvic Floor Distress Inventory Questionnaire-20 (PFDI-20) scores. The study outcomes were evaluated at pre-intervention (T0, 2 months after surgery), 3 months after surgery (T1), and 6 months after surgery (T2).

**Results:**

A total of 60 patients with POP were included in this study. For the urination function evaluation, the intervention group had a higher recovered rate than the control group at the time point of T2 (*p* = 0.038). For the EMG results, the changes of flick-max and tonic-mean values from T0 to T2 were much higher in the intervention group comparing to the control group. Corresponding to the EMG results, digital palpation showed that intervention group had a much higher proportion of patients who had elevated PFM strength. Furthermore, the intervention group also had more significant PFDI-20 score improvements compared with control group.

**Conclusions:**

Postoperative ES plus biofeedback therapy could significantly improve urination function, PFM strength, and patient’s reported QoL.

**Trial registration:**

Clinical registration number: hiCTR2000032432.

**Supplementary Information:**

The online version contains supplementary material available at 10.1007/s00384-023-04513-7.

## Introduction

Pelvic organ prolapse (POP) is defined as the descent of any vaginal compartment, which is a prevalent disorder affecting more than 40% of American post-menopausal women [1]. The patients who suffer from this disorder often complain about impaired quality of life (QoL) due to bulging symptoms and urinary, sexual, or bowel dysfunction (2). Notably, it has been reported that the symptoms of overactive bladder (OAB) syndrome have affected approximatively 88% of patients with POP (2).

Pelvic floor reconstructive surgery is the most effective and durable treatment for POP after the failure of conservative management. The surgery can excellently improve the anatomical structure of pelvic floor [2]. However, the anatomical improvement is usually not equivalent to functional rehabilitation. Frigerio et al. [3] found that the rates of persistent and de novo OAB after pelvic floor reconstructive surgery were 14.1% and 13.5%, respectively. Dray et al. [4] reported that about 77% of patients presented with at least 2 categories of symptoms after pelvic floor reconstructive surgery, with 40% of patients complained about pain and 20% of patients suffered from urination disturbance. Thus, a new strategy is needed to improve pelvic floor function for POP patients after pelvic floor reconstructive surgery.

Electrical stimulation (ES), which can excite nerves and stimulate pelvic floor muscles (PFMs) by sending mild electrical currents, is an effective method to increase voluntary pelvic muscle contractions and enhance muscle strength [5]. It has been reported that the even muscles suffering from severe impairment can be benefited from ES to perform voluntary contractions [6]. Previously, ES with or without biofeedback therapy has been proven efficacy in the treatment of pelvic floor dysfunction. For example, several clinical trials have reported that ES and/or biofeedback combined with PFM training can be used to treat postpartum urinary incontinence effectively [7, 8]. Sahin et al. [9] conducted a randomized controlled trial (RCT) and found that ES could increase motivation and treatment compliance in patients with insufficient PFM contractions. Liu et al. [10] reported recently that ES was effective to improve the rehabilitation effect of postpartum pelvic floor dysfunction. Furthermore, Yaraghi et al. [11] demonstrated in a previous study that functional ES could also improve patient’s sexual function.

Although ES with or without biofeedback is efficacy for the treatment of pelvic floor dysfunction, whether it could be used after pelvic floor reconstructive surgery to improve pelvic floor function and QoL has not been fully understood. Thus, we conducted this RCT to assess the effect of postoperative ES plus biofeedback therapy on patient rehabilitation after pelvic floor reconstructive surgery.

## Materials and methods

### Patients

Patients with POP who had received pelvic floor reconstructive surgery from March 2021 to March 2022 were included in this study.

The inclusion criteria were as follows: (1) patients with POP diagnosed according to the International Continence Society and International Urogynecological Association [12]; (2) patients with age between 40 and 70 years old; (3) patients who had received pelvic floor reconstructive surgery; (4) patients willing to voluntarily participate.

The main exclusion criteria were as follows: (1) patients who needed for postoperative radiotherapy, chemotherapy, or hormone therapy as confirmed by postoperative pathology; (2) pregnant or lactating women; (3) patients with malignant tumor, diabetes, and other serious medical diseases; (4) patients receiving treatment of urinary incontinence drugs after surgery; (5) patients with cardiac pacemaker implantation.

### Study design and treatment

This is a prospective, single-center, open-label RCT. The patients who met the inclusion criteria were randomly allocated to the intervention group and the control group at a 1:1 ratio on the basis of computer-generated random number. Randomization was performed by concealing the allocations in sequentially numbered, opaque, sealed envelopes. Blinding was not available due to the obvious proprioception of the patients receiving the intervention of ES. In order to reduce bias, the researchers who were responsible for the follow-up and evaluation were blinded to the allocation.

Patients in the control group received routine postoperative nursing care. Patients in the intervention group underwent ES plus biofeedback therapy for 10 sessions started from the 60th day (2 months) after the pelvic floor reconstructive surgery. The duration and frequency of therapy were 30 min and 3 times a week. For the ES treatments, the electric parameters were as follows: frequency: 10–50 Hz; pulse duration: 200–300 μs; on/off: 1:1; and stimulation intensity: increasing steadily from 0 mA to maximal level tolerable. The patients adopted the supine positions with 45° relaxed abducted hip and knee angles. The electrode was placed inside the vagina. For the first 3 sessions, the patients underwent ES treatments for 30 min each. From the 4th session, the biofeedback therapy was added into the therapeutic regimen. For each patient, ES treatments were conducted for 15 min, followed by 15 min of Kegel exercise with the assistance of biofeedback [5]. The instruments used in ES treatments were Vishee biostimulation feedback instrument (MLD B4, Nanjing Vishee Medical Technology Co., Ltd., Nanjing, China) and surface electromyography (EMG) electrode (Nanjing Vishee Medical Technology Co., Ltd.).

### Data collection and outcomes

Demographic and clinical data, including age, height, weight, body mass index (BMI), and the number of pregnancies and parity were collected by face-to-face interviews. POP was classified according to the international POP-Q classification. A blinded experienced physiotherapist performed evaluations of study outcomes at pre-intervention (T0, 2 months after surgery), 3 months after surgery (T1), and 6 months after surgery (T2).

The primary outcome was the recovery of urination function, which was classified into 3 levels: (1) recovered, the achievement of patient’s automatic micturition with residual urine ≤ 50 mL; (2) improved, the achievement of patient’s automatic micturition with residual urine 50–100 mL; (3) invalidated, the automatic micturition was not achieved, or residual urine was ≥ 100 mL.

The second outcomes included the PFM strength and the change of Pelvic Floor Distress Inventory Questionnaire-20 (PFDI-20) scores. The strength of PFM were evaluated via both digital palpation and EMG. PFM strength on digital palpation was assessed by a validated method and classified by the modified Oxford scale [13]. The blinded physiotherapist introduced the index and middle fingers into the vagina to palpate the puborectalis muscle during maximal contraction. The modified Oxford scale rates PFM contraction from 0 to 5: 0, no contraction; 1, minor flicker; 2, weak contraction; 3, moderate contraction; 4, good contraction; 5, strong contraction [13]. EMG was conducted following the Glazer protocols [14]. Briefly, patients were evaluated in the lithotomy position and instructed to relax the PFM. A pear-shaped vaginal manometric probe was placed into the vagina to record the electrical activity of PFM construction. To monitor unwanted muscle activation, 2 other electrodes were positioned in region of abdominal rectus muscles. The EMG for pelvic floor evaluation followed the 5-segment assessment sequence: pre-baseline for 1-min rest, recorded as mean value of initial rest (pre-mean); 5 rapid contractions with 10 -s rest period to separate them, recorded as max value of fast contractions (flick-max); 5 tonic (10 s) contractions, each separated by a 10-s rest period, recorded as mean value of 10 -s sustained contractions (tonic-mean); endurance contraction, a single, maximum, and endurance contraction until 1 min, recorded as mean value of 1-min sustained contractions (edu-mean); post baseline for 1-min rest, recorded as mean value of final rest (post-mean). The electrical activity of abdominal muscles was also recorded. The correct PFM contraction was defined as the participation of abdominal muscles < 10%.

PFDI-20 is a questionnaire designed to comprehensively evaluate the distress caused by the presence of pelvic floor dysfunction. PFDI-20 consisted of 3 scales: the Pelvic Organ Prolapse Distress Inventory (POPDI-6), the Urogenital Distress Inventory (UDI-6), and the Colorectal-Anal Distress Inventory (CRADI-8). The higher score indicated higher symptom burden [15].

### Statistical analysis

The data analysis was performed using SPSS 25.0 software (Chicago, Illinois, USA). The suitability of continuous data for normal distribution was checked by Kolmogorov–Smirnov test. The continuous data were presented as mean ± SD for those that fulfilled normal distribution, and the comparison between 2 groups was analyzed by *t*‐test. The continuous data that had not fulfilled normal distribution were presented as median and interquartile range, and the comparison was carried out by Wilcoxon rank-sum test. The categorical data were presented as percentage (%); the comparison was conducted by *χ*^2^ test or fisher exact test. The values of EMG were evaluated using general linear mixed models. *p* < 0.05 was considered as statistically significant.

## Results

**Fig. 1:**
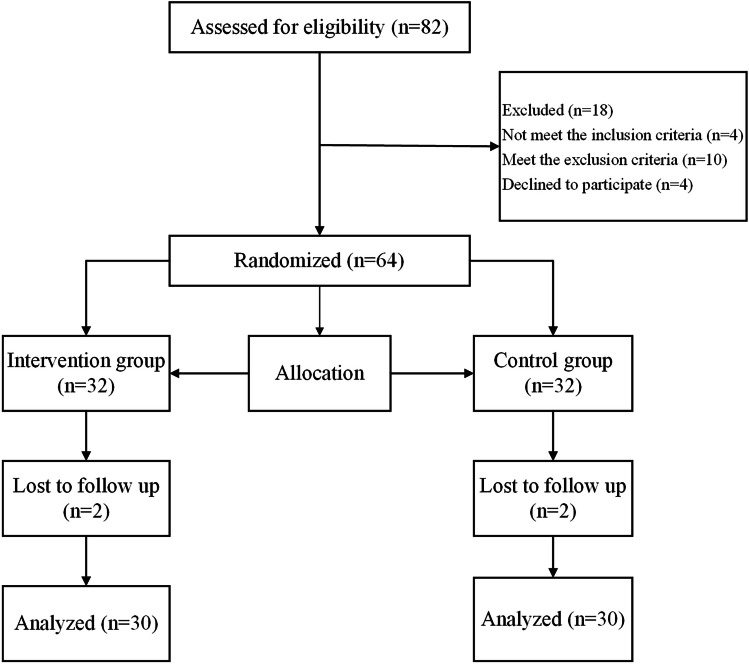
Flowchart of subject disposition in the trial

From March 2021 to March 2022, 82 POP patients who had received pelvic floor reconstructive surgery were assessed for eligibility in this study. A total of 22 patients were excluded due to various reasons, and the study was completed with 60 patients with POP. The details of the included and excluded patients are provided in Fig. 1. In the control group, 2 of the 30 cases were combined anterior and middle pelvic prolapse, and 28 cases were combined both anterior and middle pelvic prolapse; 3 cases were POP-Q II degree, and 27 cases were POP-Q III degree. In the intervention group, of the 30 cases, 1 case was pure anterior pelvic prolapse, 7 cases were combined anterior and middle pelvic prolapse, 1 case was combined middle and posterior pelvic prolapse, and 21 cases were combined anterior, middle, and posterior pelvic prolapse at the same time; 1 case was of POP-Q I degree, 5 cases were of POP-Q II degree, and 24 cases were of POP-Q III degree. The mean age of the enrolled patients was 62.80 ± 5.67 years. The demographic and physical characteristics, including age, BMI, and the number of parity, were similar between groups (*p* > 0.05, Table 1). No patient reported any adverse effects during the study period in the both groups.

**Table 1:** Clinical characteristics of patients

Characteristics	Intervention group (*n* = 30)	Control group (*n* = 30)	*p* value
Age, years, mean ± SD	62.90 ± 5.16	62.70 ± 6.23	0.893
Height, cm, mean ± SD	160.83 ± 5.51	162.67 ± 3.71	0.136
Weight, kg, mean ± SD	61.63 ± 6.69	62.10 ± 5.71	0.772
BMI, kg/m^2^, mean ± SD	23.85 ± 2.61	23.48 ± 2.13	0.547
The number of pregnancy, mean ± SD	2.07 ± 1.05	1.93 ± 1.17	0.644
The number of parity, mean ± SD	1.20 ± 0.48	1.13 ± 0.36	0.542
Smoking, *n* (%)	8 (26.67)	10 (33.33)	0.573

*BMI* body mass index, *SD* standard deviation

There was no significant difference between the 2 groups in the recovery of urination function at T0 (*p* = 0.941) and T1 (*p* = 0.038). However, at the time point of T2, the intervention group had a higher recovered rate than the control group (*p* = 0.038, Table 2).

**Table 2:** The recovery of urination function in different periods

	Intervention group (*n* = 30)			Control group (*n* = 30)			*p* value
	Recovered	Improved	Invalidated	Recovered	Improved	Invalidated	
T0	14 (46.67)	10 (33.33)	6 (20.00)	16 (53.33)	8 (26.67)	6 (20.00)	0.941
T1	24 (80.00)	4 (13.33)	2 (6.67)	19 (63.33)	6 (20.00)	5 (16.67)	0.360
T2	27 (90.00)	3 (10.00)	0 (0)	20 (66.67)	5 (16.67)	5 (16.67)	0.038

The data in the table are presented as *n* (%)

*T0* pre-intervention (2 months after surgery), *T1* 3 months after surgery, *T2* 6 months after surgery

**Fig. 2:**
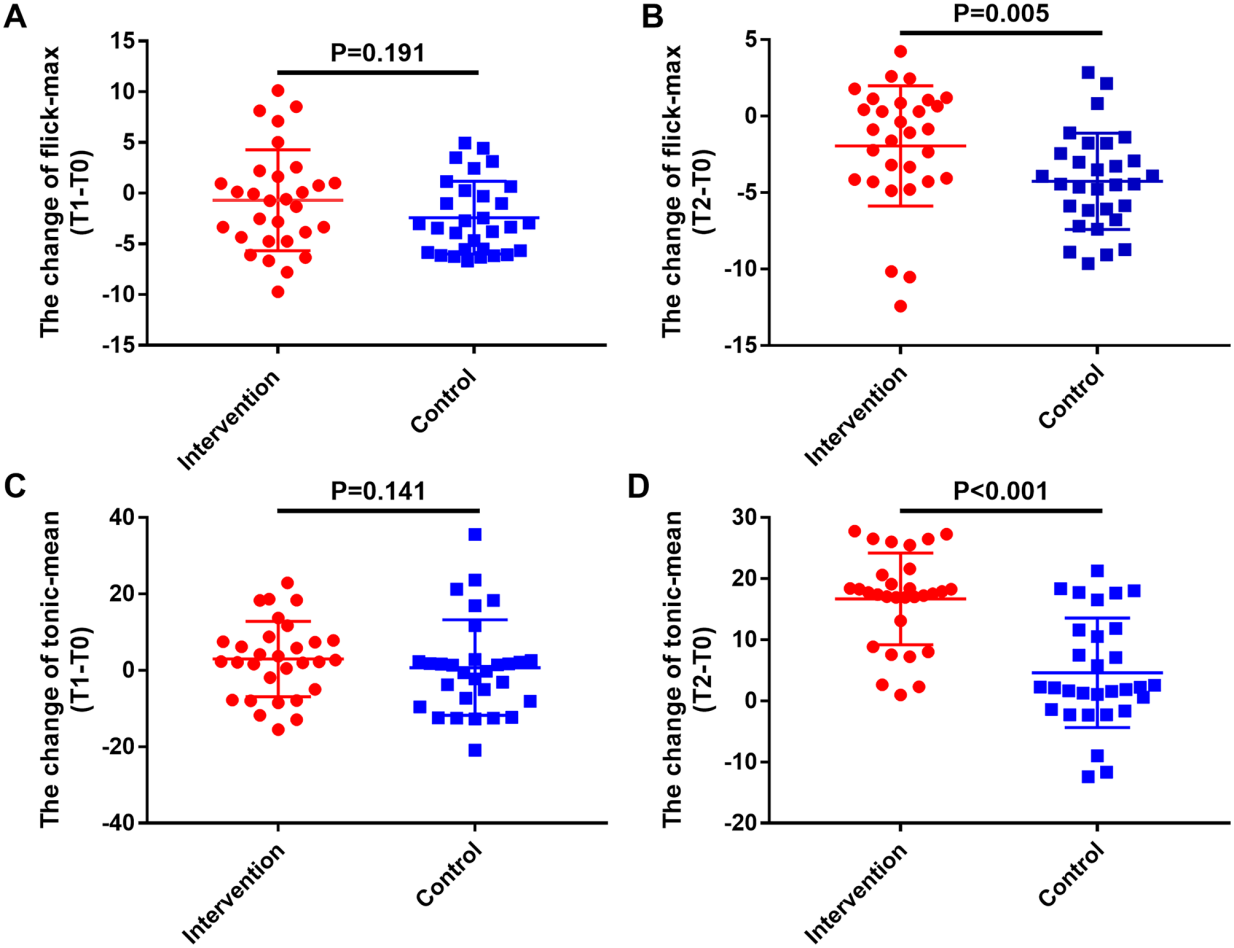
The comparisons of EMG values. **A** The change of flick-max (T1–T0); **B** the change of flick-max (T2–T0); **C** the change of tonic-mean (T1–T0); **D** the change of tonic-mean (T2–T0). EMG, electromyography

Table 3 summarized EMG values between the 2 groups during the study period. In the control group, flick-max and tonic-mean values were increased significantly at T2 compared to those measured at T0 (*p* < 0.05), but these 2 values were not significantly elevated at T1. In the intervention group, comparing to the values detected at T0, the flick-max values were significantly elevated at T1 and T2, respectively (*p* < 0.05). Furthermore, the tonic-mean values observed at T2 were much higher than those measured at T0 in the intervention group (*p* < 0.05). As shown in Fig. 2, the changes of flick-max and tonic-mean values from T0 to T1 had no significant difference between groups. However, when we calculated the changes of flick-max and tonic-mean values from T0 to T2, we found that intervention group had much higher values than control group (*p* = 0.005 and *p* < 0.001, respectively). The above results indicated that patients in the intervention group had more obvious PFM function improvement.

**Table 3:** The comparison of EMG for evaluable patients

Group	Items	T0	T1	T2
Intervention group (*n* = 30)	Pre-mean, μv	5.35 ± 0.67	4.66 ± 0.62	3.40 ± 0.45*
	Flick-max, μv	20.53 ± 1.36	25.62 ± 1.32*	38.92 ± 1.41*
	Tonic-mean, μv	16.62 ± 1.21	19.59 ± 1.11	33.31 ± 1.00*
	Variation of tonic-mean	0.26 ± 0.02	0.23 ± 0.02	0.24 ± 0.03
	Post-mean, μv	3.73 ± 0.64	3.50 ± 0.50	4.41 ± 0.50
Control group (*n* = 30)	Pre-mean, μv	6.88 ± 0.43	4.48 ± 0.62*	2.63 ± 0.29*
	Flick-max, μv	21.73 ± 1.57	25.90 ± 2.35	30.42 ± 1.73*
	Tonic-mean, μv	18.41 ± 1.16	19.14 ± 1.53	23.02 ± 1.22*
	Variation of tonic-mean	0.23 ± 0.10	0.23 ± 0.01	0.22 ± 0.01
	Post-mean, μv	4.56 ± 0.69	3.73 ± 0.57	3.37 ± 0.39

*EMG* electromyogram, *flick-max* max value of fast contractions, *post-mean* mean value of final rest, *pre-mean* mean value of initial rest, *tonic-mean* mean value of 10-s sustained contractions, *T0* pre-intervention (2 months after surgery), *T1* 3 months after surgery, *T2* 6 months after surgery

*Compared with T0, *p* < 0.05

At the time point of T1, digital palpation showed that 43.33% (13/30) of patients in control group and 73.33% (22/30) of patients in intervention group had elevated muscle strength (comparing to T0), respectively. The intervention group had a higher rate of PFM strength increasing (*p* = 0.018). Similarly, at the time point of T2, we also observed that intervention group had a much higher proportion of patients who had elevated muscle strength (27/30 vs. 18/30, *p* = 0.007), as shown in Table 4.

**Table 4:** Number of Oxford categories between the two groups at different time points

		1	2	3	4	5	Number of cases of muscle strength escalation compared to T0	*p* value
T0	Control group (*n* = 30)	0	13	14	3	0	—	
	Intervention group (*n* = 30)	0	9	18	3	0	—	
T1	Control group (*n* = 30)	0	5	17	8	0	13	
	Intervention group (*n* = 30)	0	0	10	10	10	22	0.018
T2	Control group (*n* = 30)	0	2	16	12	0	27	
	Intervention group (*n* = 30)	0	0	4	12	14	18	0.007

**Fig. 3:**
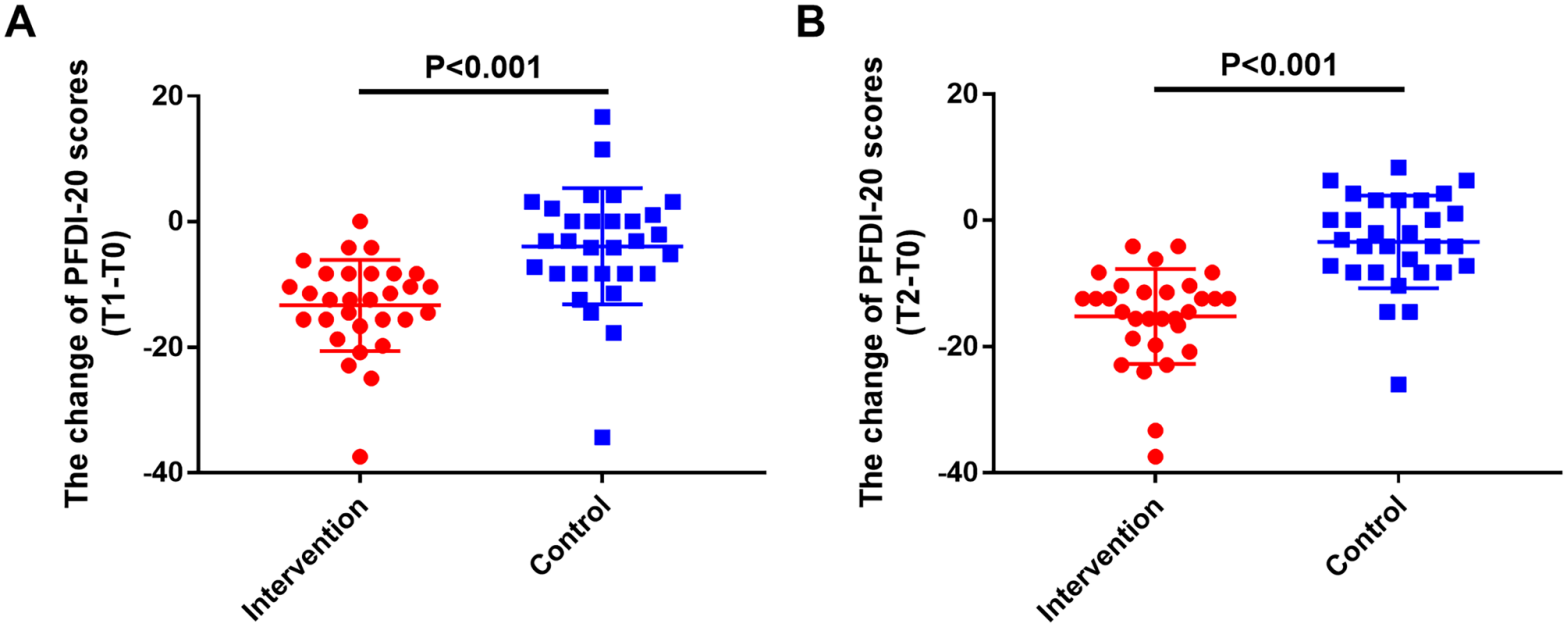
The comparisons of PFDI-20 scores. **A** The change of PFDI-20 scores (T1–T0); **B** the change of PFDI-20 scores (T2–T0). PFDI-20, Pelvic Floor Distress Inventory Questionnaire-20

PFDI-20 was used in this study to evaluate patients’ reported QoL. The median values of PFDI-20 score change from T0 to T1 for the intervention and control groups were − 12.50 and − 3.12, respectively. And the median values of PFDI-20 score change from T0 to T2 for the 2 groups were − 13.54 and − 2.60, respectively. The intervention group had more significant PFDI-20 score changes comparing to the control group (*p* < 0.05, Fig. 3 and Table S1). These results indicated that the use of postoperative ES plus biofeedback therapy could significantly improve patient’s reported QoL.

## Discussion

In the present study, we conducted a RCT to show the effect of postoperative ES plus biofeedback therapy on patient rehabilitation after pelvic floor reconstructive surgery. We found that patients who had undergone pelvic floor reconstructive surgery might be benefited from postoperative ES plus biofeedback therapy.

Pelvic floor reconstructive surgery is the most effective and durable treatment for POP. The ESTEEM trial conducted by Sung et al. [16] showed that the addition of perioperative PFM therapy to pelvic floor reconstructive surgery resulted in a small statistically significant difference in urinary incontinence symptoms compared with surgery alone. Furthermore, Pauls et al. demonstrated that pelvic floor physical therapy after pelvic reconstructive surgery improved the PFDI-20 scores and corresponding bladder symptoms, indicating pelvic floor physical therapy after surgery could improve patient’s QoL [17]. The above studies demonstrated that patients who had undergone pelvic floor reconstructive surgery might be benefited from postoperative PFM therapy or training.

For PFM training to be efficient, the ability to perform a correct contraction of these muscles is essential. Several previous studies have demonstrated that ES helps women identify and strengthen PFMs [9, 10, 18]. It is one of the effective forms for PFM training. In the present study, we evaluated the effect of postoperative ES plus biofeedback therapy on the improvement of pelvic floor function. In a previous study, low-frequency ES was found to be more effective than conventional intervention in preventing urinary retention and improving the recovery of PFM strength after radical hysterectomy for patients with cervical cancer [19]. However, a similar study conducted by Li et al. reported an unsupportive result, in which early ES after radical hysterectomy could not improve the patients voiding function, PFM strength, and QoL [20]. Different from the above studies, this study enrolled patients without malignant tumors. And we started the ES intervention only after the intra-vaginal wounds had healed at 2 months after surgery. The interval between surgery and ES intervention in this study was longer than that in Li’s trial, in which the ES treatment was given from the 7th day after surgery. Excitingly, we obtained positive results in the present study. We found that postoperative ES plus biofeedback therapy could significantly improve urination function, PFM strength, and patient’s reported QoL, comparing to the usual care.

Generally, the frequency of ES ranges from 2 to 75 Hz [20]. A preclinical study showed that ES could promote angiogenesis and nerve fiber regeneration in the detrusor and urethral sphincter, resulting in evoking bladder contraction [21]. It was reported that ES with low frequency (2–50 Hz) could promote post-operative recovery of bladder function in prostate cancer [22, 23]. In the present study, the frequency of ES was 10–50 Hz. As expected, we observed that postoperative ES plus biofeedback therapy improved the recovered rate of urination function at the time point of T2.

The effect of ES treatment on PFM strength from previous studies is uncertain. Li et al. [6] enrolled postpartum women with extremely weak muscle strength and found that ES treatment could improve the control ability of PFM contractions and elevate the muscle strength. Zhu et al. [24] reported that the patients with stress urinary incontinence (SUI) who received biofeedback ES therapy had better pelvic floor muscle endurance, strength, and coordination than those received conventional treatment. In contrast, Pereira et al. [25] conducted a pilot RCT for SUI women over 60 years, they did not discover any significance in PFM pressure between ES group and no treatment. In the present study, we found that the use of ES plus biofeedback therapy could promote PFM function recovery. For the EMG results, underwent ES plus biofeedback therapy for 10 sessions, the changes of flick-max and tonic-mean values from T0 to T2 were much higher in the intervention group comparing to the control group. Corresponding to the EMG results, digital palpation showed that intervention group had a much higher proportion of patients who had elevated PFM strength. This result indicated that patients who received postoperative ES plus biofeedback therapy had more obvious PFM strength improvement.

In clinical trials, QoL has become an important outcome measure. Previously, studies have evaluated the effect of ES on QoL improvement in SUI patients. For example, Terlikowski et al. [26] found that ES with biofeedback group had higher score of QoL questionnaire than biofeedback alone group in SUI women. Castro et al. [27] indicated that the effect of ES on the QoL was equal to PFM training in SUI patients. In the present study, we used PFDI-20, a questionnaire to evaluate the distress caused by the presence of pelvic floor dysfunction, to evaluate patient’s reported QoL before and after ES treatment. This result showed that the intervention group had more significant PFDI-20 score improvements compared with control group, indicating that the use of postoperative ES therapy significantly improved patient’s reported QoL.

The present study has several limitations. First, a long-term follow-up (6 months to 1 year or longer) was not performed. Long-term effect of postoperative ES plus biofeedback therapy on pelvic floor function after pelvic floor reconstructive surgery should be determined. Second, all patients in this study were come from a single-center. This may cause selection bias. Finally, we only used one tool to evaluate patient’s reported QoL. More tools or questionnaires are needed to adequately evaluate QoL improvements.

In conclusion, this study demonstrated that for patients with POP who had undergone pelvic floor reconstructive surgery, postoperative ES plus biofeedback therapy could significantly improve urination function, PFM strength, and patient’s reported QoL, comparing to the conventional intervention. More RCTs with sufficient sample sizes and long-term follow-up are still needed for further study.

### Supplementary Information

Below is the link to the electronic supplementary material.

Supplementary file1 (DOCX 16 KB)

## Data Availability

All data generated or analyzed during this study are included in this article. Further enquiries can be directed to the corresponding author.
