# Supplementary material for: Electrical stimulation plus biofeedback improves urination function, pelvic floor function, and distress after reconstructive surgery: a randomized controlled trial
Source: Int J Colorectal Dis. 2023 Sep 11;38(1):226. doi: 10.1007/s00384-023-04513-7 (PMC10493203; doi:10.1007/s00384-023-04513-7)
Supplement: Supplementary file 1 — Supplementary file1 (DOCX 16 KB) [file 384_2023_4513_MOESM1_ESM.docx]

Table S1 Scores on three specific PFDI-20 scales for the two groups at different time points

| Group | Items | T0 | T1 | T2 |
| --- | --- | --- | --- | --- |
| Control group (n=30) | POPDI-6 | 1.5 (1, 2) | 1 (1, 2) | 1 (1, 2) |
|  | UDI-6 | 2 (0.75, 2) | 0 (1, 2) | 1 (0.75, 2) |
|  | CRADI-8 | 1 (0, 2) | 0 (0, 1) | 0 (0, 1) |
| Intervention group (n=30) | POPDI-6 | 2 (1, 2) | 0 (0, 0.25)* | 0 (0, 0)* |
|  | UDI-6 | 1 (0.75, 2) | 0 (0, 1)* | 0 (0, 0)* |
|  | CRADI-8 | 1 (0, 2) | 0 (0, 0)* | 0 (0, 0)* |

*, compared with control group, p<0.05.
